# Simultaneous Detection of Ascorbic Acid, Dopamine, and Uric Acid Using a Novel Electrochemical Sensor Based on Palladium Nanoparticles/Reduced Graphene Oxide Nanocomposite

**DOI:** 10.1155/2020/8812443

**Published:** 2020-12-16

**Authors:** Yuyun Wei, Yangyang Liu, Zhifang Xu, Shenjun Wang, Bo Chen, Di Zhang, Yuxin Fang

**Affiliations:** ^1^Research Center of Experimental Acupuncture Science, College of Acumox and Tuina, Tianjin University of Traditional Chinese Medicine, Tianjin 301617, China; ^2^College of Pharmaceutical Engineering of Traditional Chinese Medicine, Tianjin University of Traditional Chinese Medicine, Tianjin 301617, China

## Abstract

A fresh strategy based on two-step electrochemical reduction for the fabrication of palladium nanoparticles/reduced oxide nanocomposite-modified glass carbon electrode (PdNPs/rGO/GCE) was established in this study. Field emission scanning electron microscopy (FESEM) images showed that spherical PdNPs were evenly distributed on the surface of rGO-modified electrode (rGO/GCE), and the introduction of PdNPs has no effect on the morphology of rGO. Electrochemical impedance spectroscopy (EIS) studies revealed that the conductivity of PdNPs/rGO/GCE was higher than that of rGO/GCE and bare GCE. The electrochemical performances of PdNPs/rGO/GCE sensor were investigated by cyclic voltammetry (CV), differential pulse voltammetry (DPV), and chronoamperometry using ascorbic acid (AA), dopamine (DA), and uric acid (UA) as analytes. At the optimized conditions, wide linear ranges of 0.5–3.5 mM (*R*^2^ = 0.99), 3–15 *μ*M (*R*^2^ = 0.99) and 15–42 *μ*M (*R*^2^ = 0.99), and 0.3–1.4 mM (*R*^2^ = 0.99) towards AA, DA, and UA in ternary mixture were observed, respectively. In addition to superior anti-interference capability, fast response (≤5 s), excellent reproducibility, and good long-term stability were also given by this sensor. These results suggested that PdNPs/rGO/GCE is promising for the simultaneous detection of AA, DA, and UA in practical application.

## 1. Introduction

Noble metal nanoparticles, a kind of metal nanomaterial, are often used as enhancement elements in electrochemical sensors due to their excellent electrocatalytic activity, rapid electron transfer ability, strong stability, and good biocompatibility [1–4]. Several noble metal nanoparticles, such as gold, silver, and platinum, are not suitable for commercialization due to their high cost and low availability. In contrast, palladium nanoparticles (PdNPs), an emerging noble metal nanoparticle, have been favored by researchers in recent years because of their higher abundance, lower cost, and well-resisted toxic intermediates [5–7]. However, the aggregation of PdNPs needs to be well addressed before mass application.

Researches showed that carbon nanomaterials including carbon nanotubes, carbon dots, and graphene are beneficial to the dispersion of nanoparticles due to their unique structure, large specific surface area, and high catalytic activity [3, 8]. Among them, carbon dots are more suitable for fluorescent sensors due to their unique optical properties, while carbon nanotubes are prone to serious entanglement due to their inherent strong van der Waals interaction that requires additional dispersants. Neither of them can meet people's demand for low-cost and high-performance biosensing platforms [9]. In practical application, high-quality graphene (reduced graphene oxide, rGO) is often prepared by electrochemical reduction of graphene oxide (GO), which is simple and cost-effective. The residual oxygen functional groups and the recovery of the conjugated network make rGO possess hydrophilicity and high conductivity [10–12]. Hence, rGO is considered to be the most promising carbon nanomaterials for dispersing PdNPs [13–15].

As biological molecules of great significance to human health, the abnormal changes in ascorbic acid (AA), dopamine (DA), and uric acid (UA) in human body can bring serious threats including cancer, Parkinson's disease, and leukemia [16–18]. Therefore, the development of a rapid and effective electrochemical senor for simultaneous detection of these three substances is in urgent need.

Herein, PdNPs/rGO/GCE was fabricated by facile two-step electrodeposition to detect AA, DA, and UA simultaneously. Although the preparation of PdNPs/rGO nanocomposites has been reported [19–21], most of them are prepared through a multistep complex process, which not only involves chemicals that may cause health and environmental risks but also contains long-time consumption. In addition to good catalytic activity and rapid response (≤5 s), the sensor prepared in this work also exhibited high reproducibility and stability.

## 2. Experimental

### 2.1. Reagents

AA (99.7%) was obtained from Lianxing Biotechnology (Tianjin); dopamine hydrochloride (DA, 98%) was purchased from Sigma-Aldrich (Shanghai); UA (59.0%–60.0%) and palladium chloride were provided by Ron Chemical Reagent Company (Tianjin); potassium chloride (KCl, 99.0%), potassium ferricyanide, sodium dihydrogen phosphate (NaH_2_PO_4_, 99.0%), and disodium hydrogen phosphate (Na_2_HPO_4_, ≥99.0%) were obtained from Chemical Reagent Supply and Marketing company (Tianjin); GO (99.8%) was provided by Xianfeng Nano Materials Technology (Nanjing). Phosphate buffer (PB) solution with different pH values was prepared with the mixed solution of NaH_2_PO_4_ and Na_2_HPO_4_ in a certain proportion. Ultrapure water was used throughout all measurements.

### 2.2. Apparatus

High-resolution transmission electron microscopy (HRTEM) and energy-dispersive spectroscopy (EDS) were finished on a FEI-Talos-F200X equipped with an energy-dispersive spectrometer analyzer. Field emission scanning electron microscope (FESEM) images were obtained with a Nova NanoSEM 430 (FEI, USA). Cyclic voltammetry (CV), differential pulse voltammetry (DPV), and chronoamperometry measurements were performed using a AMETEK PARSTAT 4000 electrochemical workstation (AMETEK Commercial Enterprise (Shanghai) Co., Ltd. Beijing Branch.) with a three-electrode system, while PdNPs/rGO/GCE, platinum electrode, and saturated calomel electrode (SCE) were used as a working electrode, counterelectrode, and reference electrode, respectively. KQ-600KDE high-power CNC ultrasonic cleaner was purchased from Ultrasonic Instrument Co., Ltd (Kunshan). Magnetic stirrer was provided by Ronghua Instrument Manufacturing Co., Ltd (Changzhou).

### 2.3. Fabrication of PdNPs/rGO/GCE

Before modification, the bare GCE was successively polished with 1 *μ*m, 0.3 *μ*m, and 0.05 *μ*m Al_2_O_3_ slurry and then washed ultrasonically in ultrapure water and ethanol to get a clean surface.

40 mg of GO powder was completely dissolved in 40 mL of ultrapure water under sonification for 60 min to obtain GO solution. The rGO/GCE was fabricated in GO solution by using the CV method between +1 and −1.5 V with a scan rate of 50 mV · s^−1^ for 19 cycles; after that, the modified electrode was washed with ultrapure water and dried at room temperature.

**Scheme 1:**
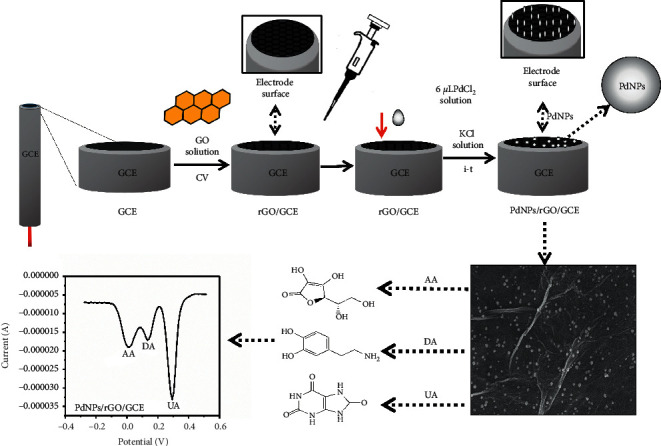
Steps for fabrication of PdNPs/rGO/GCE.

Then, 0.0035 g palladium chloride was directly added in 20 mL of ultrapure water to obtain 1 mM palladium chloride solution, and 6 *μ*L of the solution was cast on the surface of rGO/GCE. After the solution was totally dried, the electrode was immersed in 0.1 M KCl solution and then treated by applying −0.7 V for 1800 s for electrodeposition of PdNPs, and then, PdNPs/rGO/GCE was fabricated. Then, the obtained sensor was washed with ultrapure water and dried for later use (Scheme 1).

### 2.4. Electrochemical Measurements

The EIS measurements were performed from 10^−2^ to 10^5^ Hz with an amplitude of +5 V and a bias voltage of +0.24 V. DPV curves were obtained from −1.5 V to +1.5 V at an amplitude of 50 mV and a pulse width of 0.2 s. In addition, since CV technology and chronoamperometry have been used many times in this paper and the parameter settings of the same method were different, the corresponding parameter settings were introduced in the related experiments.

## 3. Results and Discussion

### 3.1. Characterization of PdNPs/rGO Nanocomposite

**Figure 1:**
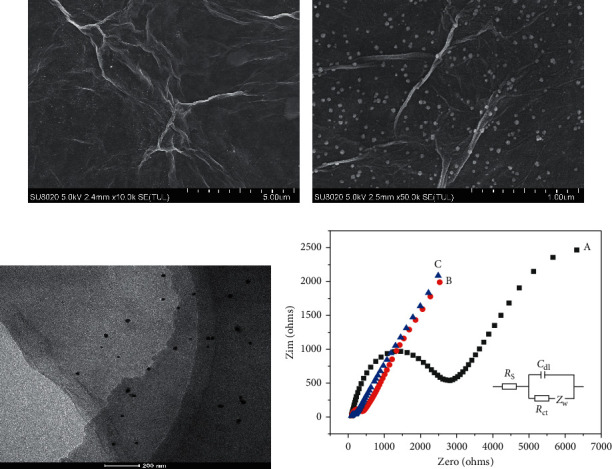
(a) FESEM characterization of rGO; (b) FESEM characterization of PdNPs/rGO nanocomposites; (c) HRETM characterization of PdNPs/rGO nanocomposites; (d) EIS characterization of bare GCE (A), rGO/GCE (B), and PdNPs/rGO/GCE (C) in 20 mM potassium ferricyanide solution containing 0.1 M KCl.

The results of FESEM and HRTEM of PdNPs/rGO nanocomposite are demonstrated in Figures 1(a)–1(c). It is evident that the synthesized rGO has a typical folded structure [22, 23]; furthermore, the PdNPs synthesized on the surface of the rGO were spherical and highly uniform in size, which not only has no effect on the morphology of the rGO but also helps to increase the surface area of the electrode (Figures 1(a) and 1(b)). From the HRTEM image (Figure 1(c)), it was obtained that the PdNPs with an average diameter less than 50 nm [24] were uniformly dispersed on the surface of rGO, which coincides with the result of Figure 1(b) and literature [25]. EDS characterization results (Figure S1) further confirm that the PdNPs/rGO nanocomposite was successfully prepared.

CV and EIS were used to examine the electrochemical behavior of PdNPs/rGO/GCE in 20 mM potassium ferricyanide solution containing 0.1 M KCl. CV curves were obtained from −0.6 V to +0.8 V with scan rate of 50 mV s^−1^ for 3 weeks. The active area of different electrodes was calculated by following Randle Sevick's equation [26]:

(1) $$\begin{array}{c} A=\frac{I_{pa}}{\left(2.69\times 10^{5}\right)}Cn^{3/2}v^{1/2}D^{1/2}, \end{array}$$

where *I*_*pa*_ is the anode peak current of different electrodes in CV experiments (Figure S2), *C* is the concentration of potassium ferricyanide solution (i.e., 20 mM), *n* is the number of electrons, *v* is the scan rate, and *D* is the diffusion coefficient. The active surface ratio of bare GCE, rGO/GCE, and PdNPs/rGO/GCE was calculated as 1 : 1.2 : 1.3, suggesting that the modification of rGO and PdNPs/rGO nanocomposite on the surface of bare GCE helps to increase the active area of the electrode that can improve the catalytic ability of bare GCE.

From Figure 1(d), the results of EIS were given in the form of Nyquist plot, in which the diameter of high-frequency semicircle part represents the resistance of charge transfer (Rct) of the electrode surface [27] and low-frequency linear part represents the diffusion process [28]. Randle's circuit (inset of Figure 1(d)) was chosen to fit the impedance data obtained. The semicircle diameter at PdNPs/rGO/GCE and rGO/GCE was much smaller than that of bare GCE (200 Ω, 400 Ω, and 3000 Ω, respectively), indicating that the modification of rGO and PdNPs/rGO nanocomposites can all help to promote the electron transfer of bare GCE. And the enhancement effect of PdNPs/rGO/GCE was greater than that of rGO/GCE, which can be attributed to the increase in the contact area between the electrode surface and the analytes after electrodeposition of PdNPs. The result of EIS was consistent with CV characterization (Figure S2).

### 3.2. Effect of pH

**Figure 2:**
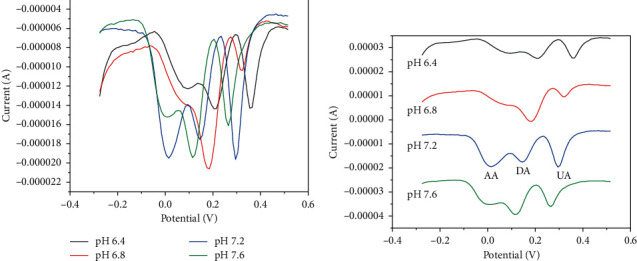
(a) DPVs of 0.75 mM AA, 30 *μ*M DA, and 1.25 mM UA at PdNPs/rGO/GCE at various pH values (black: 6.4; red: 6.8; blue: 7.2; green: 7.6); (b) DPV curves after translation.

The effect of pH of the supporting electrolyte (i.e., 0.1 M PB) on the electrochemical behavior of AA, DA, and UA was studied by DPV. As shown in Figure 2(a), with the pH values varied from 6.4 to 7.6, the peak current of AA and UA reaches the maximum at pH 7.2 (blue line), while DA reaches at pH 6.8 (red line). To clearly observe the influence of pH values on the peak potential of these three substances, the method of translation was used to separate the four curves. As can be seen from Figure 2(b), the peak potentials of AA, DA, and UA shift negatively with increasing pH values, and the peak potential differences of AA-DA and DA-UA reach the maximum at pH 7.2, suggesting that protons were involved in the oxidation reaction of AA, DA, and UA [29].

Based on above, 0.1 M PB with pH value of 7.2 was selected as the measurement medium in subsequent experiments.

### 3.3. Effect of Scan Rate

**Figure 3:**
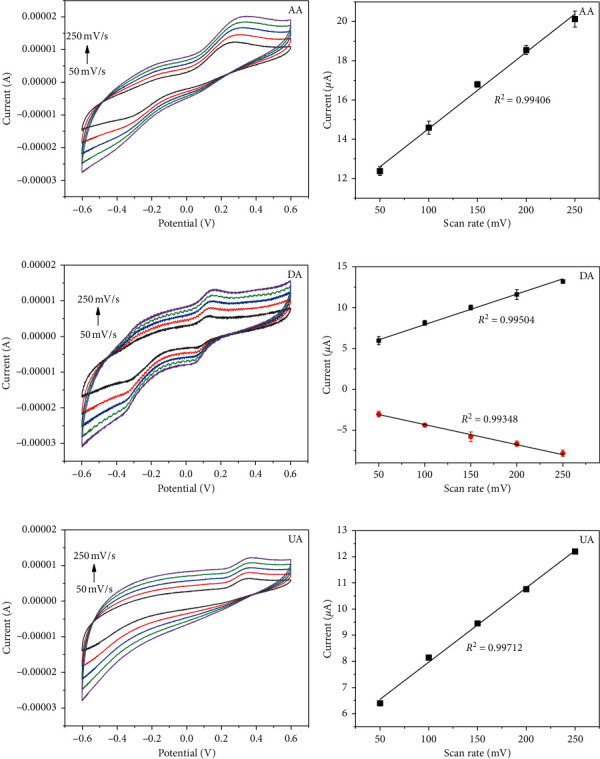
CV voltammograms of AA (a), DA (c), and UA (e) on PdNPs/rGO/GCE at 50, 100, 150, 200, and 250 mV·s^−1^ scan rates; relationship between the peak current of (b) AA, (d) DA, and (f) UA and the scan rates.

The influence of scan rate on the redox behavior of 1 mM AA, 80 *μ*M DA, and 500 *μ*M UA was studied in 0.1 M PB using CV from −0.6 V to +0.6 V for 3 weeks (Figure 3). It can be seen that the oxidation peak currents of AA, DA, and UA and the reduction peak current of DA were proportional to increasing scan rate from 50 mv · s^−1^ to 250 mv · s^−1^, and the linear regression equations were summarized as follows:

(2) $$\begin{array}{c} \text{AA}:\;I_{pa}=10.64+0.039v\;\left(R^{2}=0.99\right);\\ \text{DA}:\;I_{pa}=4.397+0.036v\;\left(R^{2}=0.99\right),\\ \;\;I_{pc}=-1.969-0.024v\;\left(R^{2}=0.99\right);\\ \text{UA}:\;I_{pa}=5.124+0.028v\;\left(R^{2}=0.99\right), \end{array}$$

where *v* is the scan rate and *R*^2^ is the correlation coefficient. The results proved that the electrochemical reaction of AA, DA, and UA was adsorption-controlled process [30, 31].

In addition, there was no obvious reduction peak in AA and UA, which may be related to the selection of detection concentration.

### 3.4. Electrochemical Detection of AA, DA, and UA

The analytical performances of PdNPs/rGO/GCE for AA, DA, and UA in 0.1 M PB (pH 7.2) were examined by DPV at the optimized conditions.

**Figure 4:**
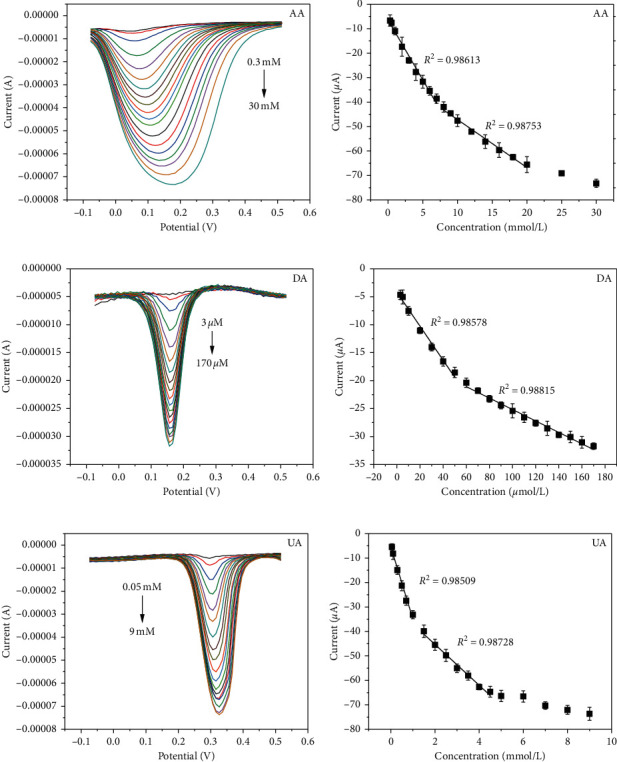
DPVs of different concentrations of (a) AA, (c) DA, and (e) UA on PdNPs/rGO/GCE in 0.1 M PB (pH 7.2); calibration plots for (b) AA, (d) DA, and (f) UA.

As can be seen from Figure 4, at PdNPs/rGO/GCE, the peak current signals of AA, DA, and UA were all gradually increased with their rising levels and achieve a good linear relationship in the concentration ranges of 0.3–7 mM and 8–20 mM, 3–50 *μ*M and 60–170 *μ*M, and 0.05–1 mM and 1.5–4.5 mM with detection limits of 0.1 mM, 1 *μ*M, and 16.67 *μ*M (S/N = 3), respectively. In addition, from Figure 4(a), it is worth noting that, with the increase in AA concentration, the peak potential of AA shifts to the right, indicating that protons have participated in the electrode reaction process of AA [32]. Meanwhile, the peak shape of AA gradually widens, which is consistent with previous studies [33–35] that may be related to the excessive concentration of AA. At low AA levels, the local AA on the electrode surface was rapidly catalyzed, and the response was fast. At high AA concentrations, a large amount of AA was adsorbed on the electrode surface, leading to the reduction of the active sites on the surface of electrode prolonging the catalytic time of PdNPs/rGO/GCE for AA, thus slowing down the catalytic process and widening the peak shape [36]. The oxidation mechanisms of AA, DA, and UA may be inferred as follows: (1) electrostatic interaction between positive DA and negative functional groups on PdNPs/rGO/GCE surface; (2) the hydrogen bond interaction between the hydroxyl groups of AA, DA, and UA and the oxygen-containing functional groups on the surface of PdNPs/rGO/GCE.

**Figure 5:**
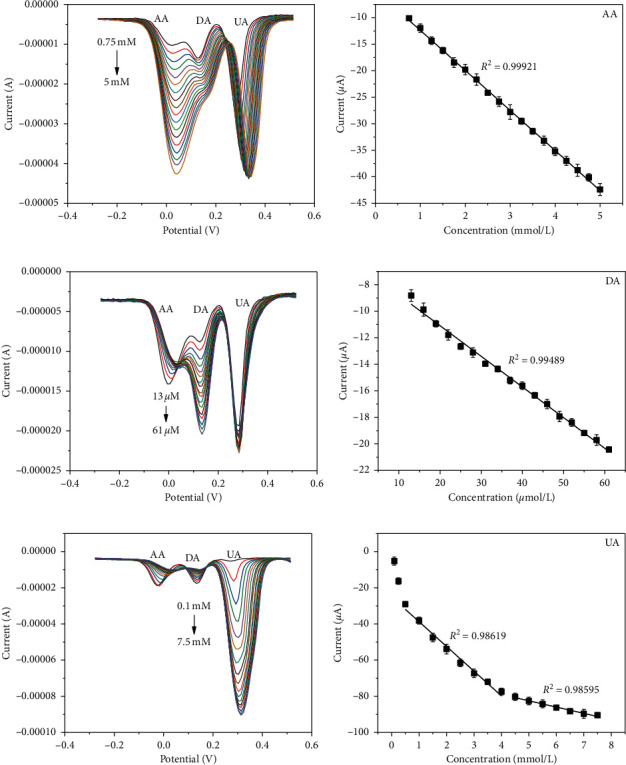
DPVs of different concentrations of (a) AA in the presence of 20 *μ*M DA and 0.5 mM UA, (c) DA in the presence of 1.5 mM AA and 0.5 mM UA, and (e) UA in the presence of 0.75 mM AA and 10 *μ*M DA. Calibration plots for (b) AA, (d) DA, and (f) UA.

**Figure 6:**
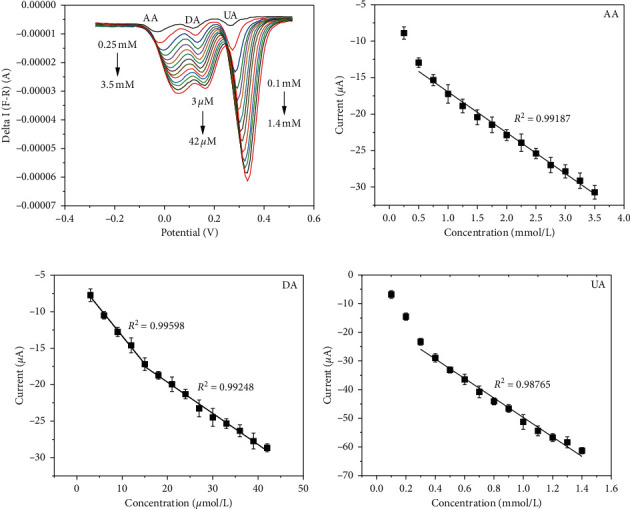
DPV curves of different concentrations of AA, DA, and UA (a); calibration plots for (b) AA, (c) DA, and (d) UA.

Figures 5 and 6 exhibit the selective and simultaneous detection results of AA, DA, and UA. It is worth mentioning that the selective detection was carried out by changing the concentrations of target species while keeping the other two substances at constant in a mixture of AA, DA, and UA. As can be observed, there exists three well-separated potential peaks corresponding to AA, DA, and UA either in selective or simultaneous detection, and the presence of the other two species did not produce significant impact on the current signal of the target analyte, suggesting that PdNPs/rGO/GCE possesses good separation capacity toward AA, DA, and UA. The detailed results are presented in Table 1. Compared with individual detection, the linear range of AA, DA, and UA under the same concentration range and the sensitivity of PdNPs/rGO/GCE toward these three analytes all produced a negligible change either in selective or simultaneous detection.

In addition, it can be observed from Figure 5(a) that, with the increase in AA concentration, in addition to the increasing AA current, the detection currents of DA and UA were also increased, which is consistent with previous literatures [28, 29, 37–41] that can be related to the adsorption of DA and UA on the electrode surface [42]. The higher concentration of AA on electrode surface continuously reacts with the oxidation products of DA and UA, resulting in the regeneration of DA and UA, thus increasing the current [43, 44]. Moreover, from Figures 4(a), 5(a), and 6(a), it is worthy to note that the oxidation voltages of AA in these three experiments were not the same, which may be related to the concentration range of AA and the interaction between AA and DA and UA [43, 44].

As shown in Table 2, although most of previous works showed higher sensitivity toward AA and UA than PdNPs/rGO/GCE, from the perspective of sensitivity toward DA and linear range, PdNPs/rGO/GCE still occupies a unique advantage in the simultaneous detection of AA, DA, and UA.

The above results showed that the developed sensor has a good application value in the detection of AA, DA, and UA.

### 3.5. Reproducibility and Stability of the Sensor

Reproducibility and stability are also crucial indicators for the evaluation of the electrochemical performances of the developed sensor.

**Figure 7:**
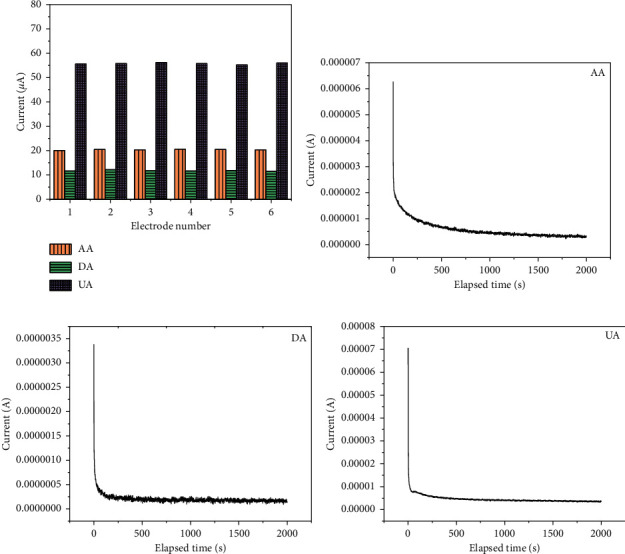
(a) Reproducibility of CQDs-rGO/GCE; stability of (b) 1 mM AA, (c) 20 *μ*M DA, and (d) 0.5 mM UA

The reproducibility of PdNPs/rGO/GCE was studied by DPV using six sensors that were prepared under the same conditions to detect 1 mM AA, 10 *μ*M DA, and 2.5 mM UA in 0.1 M PB, respectively, and the results were exhibited in the form of histogram (Figure 7(a)). The relative standard deviation (RSD) of the DPV responses of AA, DA, and UA was calculated as 0.98%, 2.08%, and 0.6%, respectively, revealing that the proposed sensor has high reproducibility.

Chronoamperometry was used to access the stability of PdNPs/rGO/GCE toward 1 mM AA, 20 *μ*M DA, and 0.5 mM UA in 0.1 M PB for 2000 s at +0.6 V. From Figures 7(b)–7(d), the current response of these three analytes reached a steady state in a short time, and the changes over a long period were negligible, which suggested that this sensor is suitable for long-term detection of AA, DA, and UA.

### 3.6. Study of Anti-Interference Ability

**Figure 8:**
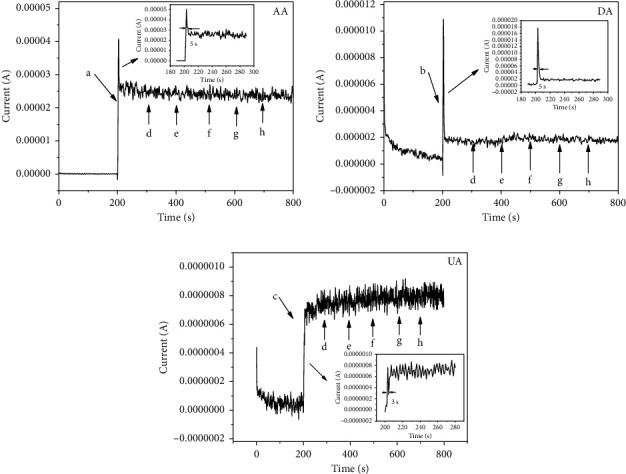
Amperometric responses of 1 mM AA (a), 50 *μ*M DA (b), and 0.1 mM UA (c) in the presence of 100-fold concentration of interferents.

Lastly, to evaluate the anti-interference ability of PdNPs/rGO/GCE, the interference of Na^+^ (d), Cl^−^ (e), Mg^2+^ (f), SO4^2−^ (g), and glucose (h) with 100-fold concentration in the detection of 1 mM AA (a), 50 *μ*M DA (b), and 0.1 mM UA (c) in 0.1 M PB was conducted by chronoamperometry at a constant potential of +0.6 V for 800 s. As shown in Figure 8, with the addition of AA, DA, and UA, the current signal of PdNPs/rGO/GCE increased rapidly with response times of 5 s, 5 s, and 3 s, respectively, and the interferents did not produce obvious effects on the current signal of AA, DA, and UA. As a result, this proposed sensor was of excellent anti-interference ability and practical application value.

### 3.7. Real Samples Detection

In order to demonstrate the applicability of the proposed method, different concentrations of AA, DA, and UA are doped into the human serum samples by the standard addition method. The DPV experimental results are shown in Table S1. The recoveries of the spiked samples were detected within the range of 96.6%–108.5%, suggesting the applicability of the prepared sensor to real samples.

## 4. Conclusions

In summary, this paper has proposed a novel approach for the synthesis of PdNPs/rGO nanocomposite by two-step CV electrodeposition; the increased surface area of as-prepared material has contributed to improve the contact probability between electrode surface and analytes, thus elevating the catalytic activity of the modified electrode, which was confirmed using CV and EIS. After optimizing the experimental conditions, the sensor showed excellent separation ability and fast response for AA, DA, and UA and has strong anti-interference ability for some common interfering substances. Besides, good reproducibility and stability were also obtained by this sensor. The above results revealed that PdNPs/rGO/GCE can be a good candidate in the sensing application of AA, DA, and UA in the future.

## Figures and Tables

**Table 1:** Analytical parameters for individual, selective, and simultaneous detection of AA, DA, and UA at PdNPs/rGO/GCE.

Analytical parameter	Analyte	Individual detection	Selective detection	Simultaneous detection
Linear range (*μ*M)	AA	300–7000, 8000–20000	750–5000	500–3500
	DA	3–50, 60–170	13–61	3–15, 15–42
	UA	50–1000, 1500–4500	500–4000, 4500–7500	300–1400
Sensitivity (*μ*A · *μ*M^−1^ · cm^−2^)	AA	0.069, 0.028	0.107	0.079
	DA	4.300, 1.443	3.254	10.893, 6.083
	UA	0.416, 0.118	0.194, 0.049	0.481

**Table 2:** Comparison of different electrodes in the simultaneous detection of AA, DA, and UA.

Electrode	pH	Linear range (*μ*M); Sensitivity (*μ*A · *μ*M^−1^ · cm^−2^)			Ref.
		AA	DA	UA	
MgO nanobelts/GCE	5.0	2.5–15, 25–150; 0.198, 0.028	0.125–7.5 7.908	0.5–3, 5–30; 2.83, 0.962	[30]
SnO_2_/chitosan/GCE	7.0	20–220; 0.127	0.1–18; 2.773	1–100; 2.391	[45]
3DGH^a^-AuNPs^b^/GCE	7.0	1.0–700; 0.217	0.2–30; 3.897	1–60; 1.703	[46]
AuNPs^b^@MoS_2_ nanosheets/GCE	4.0	12–800; 0.481	10–300; 0.979	8–900; 0.465	[47]
Pd_3_Pt_1_^c^/PDDA^d^-rGO/GCE	7.4	40–1200; 0.359	4–200; 0.639	4–400; 0.498	[48]
CB^e^/GCE	7.0	1.91–37.8; 0.214	0.599–11.8; 1.570	1.01–14; 0.680	[49]
Pt@NP-AuSn^f^/CFP^g^	7.0	200–1200; 0.0004	0.5–10; 0.0017	25–500; 0.0003	[50]
PdNPs/rGO/GCE	7.2	500–3500; 0.079	3–15, 15–42; 10.893, 6.083	300–1400; 0.481	This work

a: three dimensional graphene hydrogel; b: gold nanoparticles; c: Pd-Pt bimetallic nanoparticles; d: poly(diallyldimethylammonium chloride); e: nanostructured carbon black; f: Pt nanoparticle-modified nanoporous AuSn; g: Ni-buffered flexible carbon fiber paper.

## Data Availability

The generated or analyzed data used to support the findings of this study are included within the article.

## Supplementary Materials

Supplementary Materials Figure S1: EDS characterization of PdNPs/rGO nanocomposites. Figure S2: CV characterization of bare GCE (a), rGO/GCE (b), and PdNPs/rGO/GCE (c) in 20 mM potassium ferricyanide solution containing 0.1 M KCl. Table S1: detection of AA, DA, and UA in real samples (*n* = 3).
