# Supplementary material for: Simultaneous Detection of Ascorbic Acid, Dopamine, and Uric Acid Using a Novel Electrochemical Sensor Based on Palladium Nanoparticles/Reduced Graphene Oxide Nanocomposite
Source: Int J Anal Chem. 2020 Dec 16;2020:8812443. doi: 10.1155/2020/8812443 (PMC7759412; doi:10.1155/2020/8812443)
Supplement: Supplementary Materials — Figure S1: EDS characterization of PdNPs/rGO nanocomposites. Figure S2: CV characterization of bare GCE (a), rGO/GCE (b), and PdNPs/rGO/GCE (c) in 20 mM potassium ferricyanide solution containing 0.1 M KCl. Table S1: detection of AA, DA, and UA in real samples (n = 3). [file 8812443.f1.docx]

**Supporting information：**


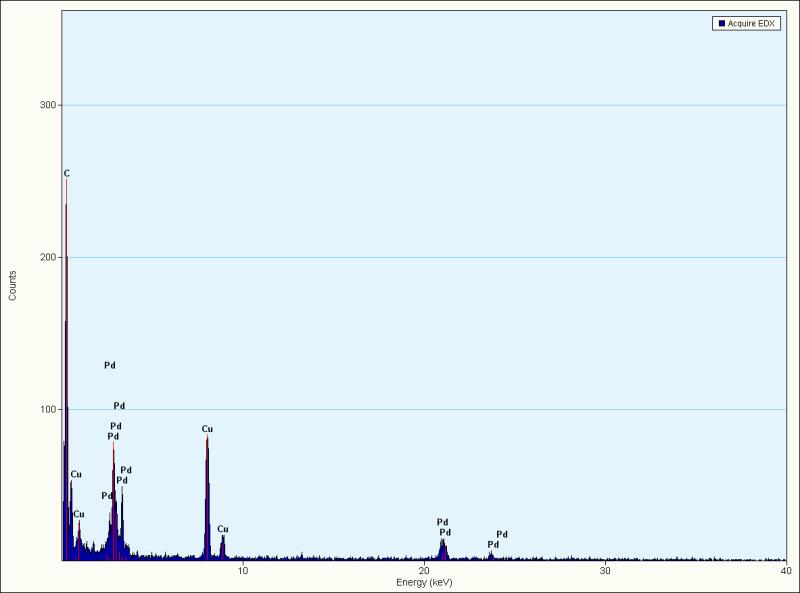


Fig. S1 EDS characterization of PdNPs/rGO nanocomposites.





Fig. S2 CV characterization of bare GCE (a) , rGO/GCE (b) and PdNPs/rGO/GCE (c) in 20 mM potassium ferricyanide solution containing 0.1 M KCl.

Table S1 Detection of AA, DA and UA in real samples (n=3).

| Samples | Analyte | Spiked (μM) | Found (μM) | Recovery (%) |
| --- | --- | --- | --- | --- |
| 1 | AA | 500 | 485.23 | 97 |
|  | DA | 20 | 21.7 | 108.5 |
|  | UA | 300 | 316.86 | 105.6 |
| 2 | AA | 600 | 588.65 | 98.1 |
|  | DA | 30 | 28.98 | 96.6 |
|  | UA | 400 | 409.75 | 102.4 |
| 3 | AA | 700 | 688.35 | 98.3 |
|  | DA | 40 | 40.85 | 102.1 |
|  | UA | 500 | 493.55 | 98.7 |
